# PREDICTING EFFECT AND EVALUATING COST-EFFECTIVENESS OF A FAMILY INTERVENTION AFTER ACQUIRED BRAIN OR SPINAL CORD INJURY: A RANDOMIZED CONTROLLED TRIAL

**DOI:** 10.2340/jrm.v58.44691

**Published:** 2026-02-11

**Authors:** Karoline Yde ANDERSEN, Frederik Have DORNONVILLE DE LA COUR, Mia Moth WOLFFBRANDT, Fin BIERING-SØRENSEN, Juan Carlos ARANGO-LASPRILLA, Pernille Langer SOENDERGAARD, Anne NORUP

**Affiliations:** 1 Neurorehabilitation Research and Knowledge Centre, Copenhagen University Hospital – Rigshospitalet, Glostrup; 2 Department of Neuroscience, University of Copenhagen, Copenhagen; 3 The Elsass Foundation, Charlottenlund; 4 Department of Clinical Medicine, University of Copenhagen, Copenhagen; 5 Department of Brain and Spinal Cord Injuries, Rigshospitalet, Glostrup, Denmark; 6 Department of Cell Biology and Histology, University of the Basque Country UPV/EHU, Leioa; 7 IKERBASQUE, Basque Foundation for Science, Bilbao, Spain, and; 8 Neurorehabilitation-CPH, City of Copenhagen, Hellerup, Denmark

**Keywords:** economic evaluation, health economic analysis, neurorehabilitation, stroke, family intervention

## Abstract

**Objective:**

To predict treatment response of a family intervention and investigate its cost- effectiveness from a healthcare payer perspective.

**Design:**

Explorative predictor analyses and trial-based economic evaluation.

**Subjects/Patients:**

Participants (*n*=157) were enrolled 4 months to 2 years after discharge from a specialized neurorehabilitation unit.

**Methods:**

Injury type, time since injury, delivery format, and relationship (individual with injury vs family member) were explored as predictors of effect using linear mixed-effect regression. Cost-effectiveness was analysed from a healthcare payer perspective, with incremental cost based on delivery. Incremental health effect was reported for measures on mental health and anxiety and depression symptoms.

**Results:**

The 4 predictors had negligible to small effects on the treatment response. Incremental cost for the family intervention was estimated at €798.16 (CI: €700.9; €895.5). Incremental health effect was estimated at 5.64 (CI: 2.71, 8.56) points on the Mental Component Summary at 2 months’ follow-up. At a willingness-to-pay threshold of €300, the probability of the intervention being cost-effective was 99.8% for the Mental Component Summary.

**Conclusion:**

The predictors showed no or little effect on the treatment response, and the cost-effectiveness analysis showed the probabilities of the intervention being cost-effective from a health payer perspective.

## LAY ABSTRACT

Acquired brain or spinal cord injuries present significant challenges not only for the affected individual but also for their family. This study evaluated a manualized family intervention consisting of 8 sessions facilitated by neuropsychologists, in which the entire family participated. The aim was to identify factors that might explain variability in treatment outcomes and to assess the cost-effectiveness of the intervention. A total of 157 participants were included: 73 individuals with an injury and 84 family members. Injury type, time since injury, delivery format, and participant role (injured individual vs family member) had little to no influence on the intervention outcomes. Moreover, the intervention demonstrated a high probability of being cost-effective, taking into account both the direct costs of the intervention and indirect costs related to participants’ time and transportation.

Individuals with acquired brain injury (ABI) and spinal cord injury (SCI) often face challenges in daily life, not only for the individual who sustained the injury, but also for their family members (1, 2). ABI encompasses a broad spectrum of neurological conditions occurring after birth, including both traumatic and non-traumatic brain injuries, often resulting in long-lasting sequelae in several domains (3). SCI can result in partial or complete loss of motor, sensory, and autonomic functions below the level of injury on the spinal cord (4). These injuries can impact long-term family functioning, causing emotional burden, mental health issues, and reduced quality of life (2, 5, 6). The reliance on family members for informal care can lead to caregiver burden, family imbalance, and relationship strain (1, 6–8). To address these challenges, the Family Intervention after Acquired Brain or Spinal Cord Injury investigated the effectiveness of an 8-week manual-based family intervention to improve mental health-related quality of life and reduce caregiver burden (9). The 8 weekly sessions are 90-min long and are facilitated by trained neuropsychologists. The programme combines psychoeducation, cognitive-behavioural therapy, and family therapy techniques to improve communication, emotional management, problem-solving, and overall family cohesion. The intervention is tailored to address challenges unique to families experiencing life changes after traumatic injury. The clinical effectiveness of the intervention has already been determined in a previous paper, where the primary outcomes showed significant improvements in mental health-related quality of life and reductions in caregiver burden for the Family Intervention Group (FIG) compared with the Psychoeducational Group (PEG) (9). Due to the COVID-19 pandemic, the family intervention was initially delivered in person and later transitioned to videoconferencing. Although the family intervention was effective at the group level, it remains unclear whether certain individuals benefited more than others or whether the videoconferencing format was as effective as the in-person format. Identifying predictors of outcomes is essential for tailoring the intervention and enhancing its impact during implementation.

In addition, the economic aspect plays a crucial role for healthcare decision-making in the appraisal and selection of new interventions, services, and technologies (10). ABI and SCI have significant economic impacts, leading to higher healthcare costs, higher divorce rates, and an increased risk of job loss for both affected individuals (11, 12) and their family members (13). Despite these economic implications, few studies have examined the cost-effectiveness of rehabilitation within the field of ABI and SCI (14, 15). A review by Mancuso et al. (16) revealed that most existing studies on economic evaluations of ABI focus on treating language disorders, while there is limited research on general rehabilitation programmes. Understanding cost-effectiveness is crucial for healthcare services seeking to optimize rehabilitation and allocating resources. Furthermore, identifying factors associated with effectiveness of these interventions can help tailor services for the specific rehabilitation need.

This study aims to explore predictors of treatment response to the family intervention and analyse the costs associated with the trial relative to its effects, by examining:

1. the effect on treatment outcome by 4 predictors: (*i*) injury type (ABI vs SCI); (*ii*) time since injury; (*iii*) delivery format (in-person vs online); and (*iv*) relation (individual with injury vs family member);
2. the cost-effectiveness of the family intervention from a healthcare payer perspective.

## METHODS

The family intervention was a two-arm randomized controlled trial (RCT) conducted in Denmark between October 2018 and June 2021. Individuals with ABI or SCI and their family members were enrolled 4 months to 2 years after discharge from a specialized neurorehabilitation unit. Inclusion criteria were age ≥18 years, ability to understand and speak Danish, and cognitive capacity to participate in the study. Participants were excluded if they had been previously diagnosed with a neurological or psychiatric disorder, had experienced violence in their family, or were struggling with substance abuse at the time of inclusion. All participants provided written informed consent in accordance with the Helsinki Declaration. Further details concerning the recruitment, randomization, and inclusion/exclusion for the main effectiveness analysis are provided by Soendergaard et al. (9). Follow-up outcomes were assessed 2 and 8 months after baseline. The RCT study was reported to the Danish Data Protection Agency (journal no. P-2021-603) and to the Committees on Health Research Ethics on the Capital Region of Denmark (journal no. H-1801 4858). The RCT was registered on 24 January 2019 at ClinicalTrials.gov, identifier: NCT03814876.

### Intervention

The sample consisted of 157 participants with ABI (*n* = 53) or SCI (*n* = 20) and their family members (*n* = 84). Participants were randomly assigned to either the FIG (*n* = 74) or the PEG (*n* = 83). Both the FIG and PEG were a supplement to treatment-as-usual (TAU), as there is no systematically offered family-involving intervention for individuals with ABI or SCI and their family members in Denmark. The FIG received an 8-week manual-based intervention consisting of 90-min sessions delivered to each family individually by a trained neuropsychologist. The intervention incorporated strategies from rehabilitation psychology, cognitive behavioural therapy, and family therapy to enhance well-being and psychological functioning (17). The families received between-session tasks. An overview of the 8 sessions is outlined in Soendergaard et al. (9). The PEG received an optional single 2-h psychoeducation session on the consequences of an injury to the brain or spinal cord. This was administered to groups or individual families by an experienced neuropsychologist. Initially, the FIG and PEG were delivered in person at a specialized hospital in Copenhagen. However, due to the COVID-19 pandemic, both groups transitioned to videoconferencing during lockdowns from March 2020, with some families receiving both in-person and videoconferencing sessions. In FIG, 11 families received videoconferencing, and in PEG, 7 families.

### Cost-effectiveness analysis

This study presents a health economic trial-based evaluation over 8 months, from a healthcare payer perspective, with partial extension of a social perspective, which included costs for participants’ time use and transportation, estimated for both the individual with an injury and their family members. The health-care payer perspective focuses on the costs directly related to the intervention (18). Due to limited trial data, a broader perspective with additional costs and savings to the healthcare system or society was not considered in the analysis. The analysis adheres to the Consolidated Health Economic Evaluation Reporting Standards (CHEERS) (19).

### Cost outcomes

Resources associated with the FIG and PEG were registered during the RCT, and costs were determined in October 2024. All costs were calculated for each participant/family and restricted to an 8 months’ time horizon, matching the follow-up period of the trial. A micro-costing approach was used to calculate the costs of the interventions. This methodological approach is based on granular and detailed cost data, which are crucial for robust economic analyses (20). Resources were therapist time used for session and preparation, administration, materials for the session, overhead, participants’ time use, and transportation cost (Appendix S1). Costs were recorded in 2020 Danish Kroner (DKK) and reported in 2020 Euro (€1=DKK7.45); 2020 prices were selected as most of the costs incurred in that year. Discounting was not applied due to the short time horizon of 8 months.

The cost of staff time was based on the average gross salary paid to a psychologist and a research nurse, assuming an effective working month of 94 h (21, 22). Staff time included session time with the addition of a preparation time of 30 min per session, and administration of participant inquiries via phone or email assumed to be 10 min per contact. A standard overhead rate of 21%, as per Copenhagen University Hospital – Rigshospitalet, was applied to account for non-attributable costs.

The average costs incurred by participating families included transportation expenses and time use associated with attending intervention session(s) and time of transportation. These costs were based on a registered data log completed by a research nurse. Transportation costs were based on individual round-trip distance in kilometres to and from in-person session(s), assuming family members travelled together, as most family members were living with the injured individual, 89% in the FIG and 82% in the PEG. Travel costs were valued using Danish kilometre allowance in 2020 (23). Further details regarding costs and source are available in Appendix S1.

### Health outcomes

The effectiveness of the family intervention was measured by self-reported questionnaires, completed by all participants at baseline, 2 months’ follow-up, and 8 months’ follow-up. The primary outcome for this cost-effectiveness analysis included: (*i*) Mental-health-related quality of life, measured by the Mental Component Summary (MCS) of the 36-item Short-Form Health Survey (SF-36v2). The MCS is a sum score of mental health subscales: Vitality, Social Functioning, Role Emotional, and Mental Health. The score ranges from 0 to 100, with higher scores indicating better health (24); (*ii*) the Generalized Anxiety Disorder (GAD-7) is a 4-point Likert-type scale ranging from 0 (not at all) to 3 (nearly every day). The scale contains 7 items, with scores ranging from 0 to 21, with higher scores indicating more anxiety symptoms (25); and (*iii*) the Patient Health Questionnaire depression scale (PHQ-9) is a 4-point Likert-type scale ranging from 0 (not at all) to 3 (nearly every day). The scale consists of 9 items, with scores ranging from 0 to 27, with higher scores indicating more depression symptoms (26).

### Incremental cost-effectiveness

Cost-effectiveness was measured as the additional cost of gaining 1 additional point on MCS, GAD-7, and PHQ-9. Incremental cost-effectiveness ratios (ICER) were computed as the difference between FIG and PEG in cost, divided by the difference between groups in health outcome measures gained from baseline to 2 months’ follow-up and from baseline to 8 months’ follow-up.

### Statistical analysis

Analyses were conducted on an intention-to-treat basis. The explorative predictor analyses were conducted for the FIG only, using the same approach as the RCT study (9), with linear mixed-effect regression models with a fixed effect of time and random intercepts for individuals and families. To evaluate the predictors, a main effect and a predictor-by-time effect was added to the model. Effects at the 2 months’ and 8 months’ follow-up were explored in separate models. Four predictors were examined separately: time since injury (grouped by the median), type of injury (ABI or SCI), delivery format (in-person or online sessions), and relation (individual with injury or family member). Predictors were chosen based on previous research suggesting that time since injury, type of injury, and relationship can affect burden and emotional distress (27). Furthermore, we wanted to investigate the format, as this was changed during the study. Partially standardized coefficients were calculated by standardizing the dependent variable. The size of the predictor-by-time effect was computed using Cohen’s incremental *f* ^2^ and interpreted as small at a value of 0.02, medium at a value of 0.15, and large at a value of 0.35 (28, 29).

The analyses for the cost-effectiveness part of the study were based on the same approach as for the RCT study (9). Between-group differences were analysed using linear mixed-effect regression models for each outcome measure, with random effects for individuals and families. Fixed effects included group allocation, time, and an interaction effect of group by time. Parameters were estimated using maximum likelihood estimation. In accordance with recommendations, missing data were handled by imputation: on the MCS from SF-36v2 were imputed if 7 or more item scores were available, using the Missing Score Estimation from Quality Metric Optum (30). For the GAD-7 and PHQ-9, missing data were imputed by the mean score if 1 item was missing on GAD-7, and if up to 2 items were missing on PHQ-9 (25, 31).

The ICERs were estimated based on a non-parametric bootstrap resampling procedure, generating 10,000 bootstrap samples by randomly resampling observations, and 2.5 and 97.5 percentiles were interpreted as confidence limits. The 10,000 bootstrapped samples were used to generate cost-effectiveness acceptability curves (CEAC) to visualize uncertainty by determining the cost-effectiveness at a given willingness-to-pay (WTP) threshold. The probability of the FIG being cost-effective was evaluated from a healthcare payer perspective with addition of the participants’ cost using a threshold from €0 to €1,000.

For the main analysis, best available prices were used for costs. Deterministic sensitivity analyses were performed to determine the robustness of study findings unit costs. Alternative estimates were used at a pessimistic and optimistic scenario analysis of costs units. Further details for the construct of the sensitivity analyses are provided in Appendix S2.

All analyses were conducted in R version 4.4.1 (R Foundation for Statistical Computing, Vienna, Austria) using the lme4 (32) and emmeans (33) packages. Data are not openly accessible due to Danish data regulations.

## RESULTS

A total of 157 participants were included in the main analysis. Table I summarizes participant characteristics at baseline before randomization. There were no statistically significant differences between group characteristics at baseline. For the FIG, 14 participants discontinued the sessions, and dropped out between session 2 and session 6, and for the PEG, 14 did not participate in the optional psychoeducation session. The mean age in the FIG was 53 years old and in the PEG 50 years old. In the FIG, 47% used prescribed medicine and in the PEG 52%. Most participants received rehabilitation in hospital, 69% in FIG and 84% in PEG or in the municipality, 68% in FIG and 69% in PEG.

**Table I:** Baseline characteristics of participants in the family intervention group (FIG) and the psychoeducational group (PEG)

Factor	*n*	FIG (n=74) Individual with injury=35 Family members=39	*n*	PEG (n=84) Individual with injury=38 Family members=45	*p*-value^a^
Sex, *n* (%)					
Female	74	35 (47)	83	42 (51)	0.80
Male		39 (53)		41 (49)	
Age, mean (SD)	74	53 (16.9)	83	50 (14.4)	0.23
Range		21; 84		18; 80	
Type of injury, *n* (%)					
TBI	35	13 (37)	38	11 (29)	0.37
Stroke		12 (34)		13 (34)	
SCI		9 (26)		11 (29)	
Other^b^		1 (3)		3 (8)	
Time since injury^c^, mean (SD)	35	16 (8.9)	38	15.6 (8.3)	0.87
Range		6; 49		6; 39	
Education, *n* (%)					
Low	73	24 (32)	83	32 (39)	0.44
High^d^		46 (62)		50 (60)	
Other		3 (4)		1 (1)	
Work status, *n* (%)					
Full-time occupation	72	32 (43)	81	43 (52)	0.79
Student		5 (7)		5 (6)	
Homemaker		2 (3)		2 (2)	
Unemployed		4 (5)		2 (2)	
Retired		20 (27)		17 (20)	
Sick leave		9 (12)		12 (14)	
Civil status, *n* (%)					
In a relationship	74	69 (93)	81	67 (81)	0.08
Single		5 (7)		11 (13)	
Other				2 (2)	
Living status, *n* (%)					
Living with partner or other	74	72 (97)	82	74 (89)	0.10
Living alone		2 (3)		8 (10)	
Treatment with psychologist, yes, *n* (%)					
Before baseline	74	19 (26)	83	32 (39)	0.09
Currently	73	7 (10)	82	11 (13)	0.62
Medication use, yes, *n* (%)	73	35 (47)	83	43 (52)	0.75
Rehabilitation, yes, *n* (%)					
Hospital	35	24 (69)	38	32 (84)	0.17
Municipality	35	24 (68)	38	26 (68)	1
Private	35	7 (20)	38	3 (8)	0.18
Other	35	1 (3)	38	2 (5)	1

a A *t*-test was used for continuous variables, Fisher’s exact test was used for categorical variables;

b tumour, heart attack, and haematologic disease;

c reported in months;

d high level of education indicates a college or university degree.

TBI: traumatic brain injury; SCI: spinal cord injury; SD: standard deviation.

### Exploratory predictor analyses

Table II summarizes the results of the exploratory predictor analyses. The 4 predictors showed negligible to small effect sizes on the MCS score at the 2 time points with *p*-values ranging from 0.17 to 0.91 at 2 months’ follow-up and from 0.13 to 0.94 at 8 months’ follow-up. Type of injury was associated with a small effect size at 8 months’ follow-up, *f* ^2^ = 0.046, *p* = 0.13. From baseline to 8 months’ follow-up, individuals with ABI and their family members gained 3.9 points less on the MCS compared with SCI.

**Table II:** Exploratory multilevel modelling analysis for the Mental Component Scale (MCS)

Factor	Two months’ follow-up					Eight months’ follow-up				
	Est. (SE)	95% CI	*p*-value^a^	Std. est. (SE)	*f* ^2 b^	Est. (SE)	95% CI	*p*-value^a^	Std. est. (SE)	*f* ^2 b^
Type of injury x Time Reference group: SCI	0.65 (2.08)	–3.43, 4.73	0.755	0.05 (0.17)	0.002	–3.91 (2.53)	–8.87, 1.04	0.127	–0.31 (0.20)	0.046
Time since injury x TimeReference group: ≥ median	–0.22 (2.01)	–4.15, 3.71	0.913	–0.02 (0.16)	–0.002	–0.21 (2.51)	–5.13, 4.72	0.935	–0.02 (0.20)	–0.001
Delivery format x TimeReference group: in-person	–2.78 (2.02)	–6.75, 1.18	0.173	–0.22 (0.16)	0.013	–0.37 (2.5)	–5.32, 4.58	0.884	–0.03 (0.20)	–0.001
Relation x Time Reference group: injured individual	0.77 (1.98)	–3.11, 4.66	0.698	0.06 (0.16)	0.002	0.28 (2.46)	–4.55, 5.11	0.909	0.02 (0.20)	–0.001

a Satterthwaite’s method;

b Cohen’s incremental *f*^2^.

SCI: spinal cord injury; SE: standard error; CI: confidence interval.

### Health outcome

Table III summarizes the estimated change in outcome scores for the FIG and PEG, and the incremental effect of the scores. The incremental effect showed the largest between-group differences for the MCS score at baseline to 2 months’ follow-up. For the GAD-7 and PHQ-9 score, it was from baseline to 8 months’ follow-up.

**Table III:** Effect of the family intervention group (FIG) and the psychoeducational group (PEG) on clinical outcomes

Outcome	Estimated change in score, (95% CI)		Incremental effect, (95% CI) ∆E^FIG - PEG^
	FIG	PEG	
MCS, baseline to M2	4.96^***^ (2.55, 7.38)	–0.67 (–2.95, 1.61)	5.64^***^ (2.71, 8.56)
MCS, baseline to M8	4.22^***^ (1.74, 6.69)	–0.37 (–2.68, 1.93)	4.59^**^ (1.61, 7.57)
GAD-7, baseline to M2	–1.40^**^ (–2.42, –0.38)	–0.34 (–1.31, –0.64)	–1.06 (–2.30, 0.18)
GAD-7, baseline to M8	–1.54^**^ (–2.59, –0.48)	0.24 (–0.74, 1.21)	–1.77^**^ (–3.04, –0.51)
PHQ-9, baseline to M2	–0.79 (–1.72, 0.15)	0.53 (–0.37, 1.44)	–1.32^*^ (–2.47, –0.17)
PHQ-9, baseline to M8	–1.20^*^ (–2.17, –0.23)	0.28 (–0.62, 1.68)	–1.48^**^ (–2.64, –0.32)

MCS: Mental Component Summary; GAD-7: Generalized Anxiety Disorder; PHQ-9: Patient Health Questionnaire depression scale; CI: confidence interval;

* *p* < 0.5;

** *p* < 0.01;

*** *p*<0.001.

### Cost outcome

The incremental cost between FIG and PEG showed that the family intervention was more costly, both when considering the intervention cost and participants’ cost separately (Table IV). The total cost per participant was €1,064.44 for the FIG and €266.27 for the PEG (Table IV).

**Table IV:** Health system cost and productivity losses for the family intervention group (FIG) and the psychoeducational group (PEG), per participant

Cost	Mean cost, (SE)		Incremental cost, (95% CI) ∆C^FIG - PEG^
	FIG	PEG	
Healthcare provider costs	€535.52 (21.35)	€120.13 (5.56)	415.38 (372.1; 458.6)
Participants costs	€528.91 (31.75)	€146.14 (13.74)	382.76 (315.0; 450.6)
Total cost	€1,064.44 (46.97)	€266.27 (16.06)	798.16 (700.9; 895.5)

SE: standard error; CI: confidence interval.

The total cost of the interventions, without participants’ cost, were €39,629 for the FIG and €9,971 for the PEG. This includes costs associated with delivery of the sessions both in-person and online. Total cost for participants was based on time use and utilities for transportation and was estimated at €39,139 for the FIG and €12,129 for the PEG. For a more detailed description of the costs see Appendix S1.

During the intervention period, the participating families had a mean attendance of 7.06 (SD: 2.01) sessions in the FIG and 0.84 (SD: 0.37) for the PEG. For the PEG, 18 sessions were with individual families and 6 sessions were with 2 or more families attending together. On average, round-trip distance to and from session(s) was 41.4 kilometres for the FIG and PEG; both groups attended sessions at the same hospital.

### Cost-effectiveness

**Fig. 1:**
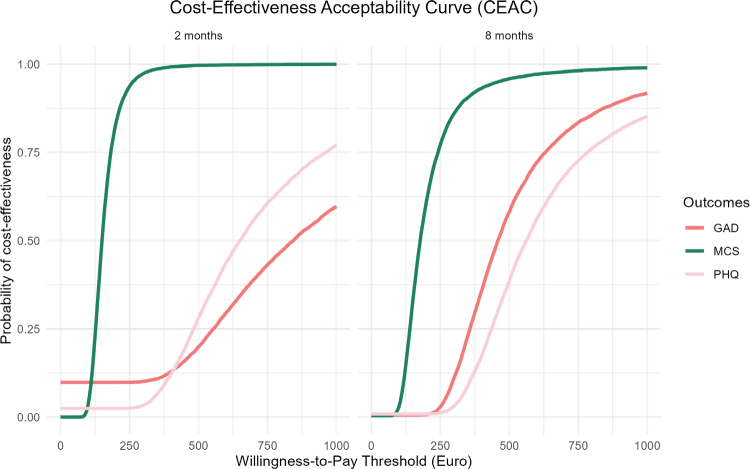
Cost-effectiveness acceptability curve: probability of the family intervention being cost-effective. MCS: Mental Component Summary; GAD-7: Generalized Anxiety Disorder; PHQ-9: Patient Health Questionnaire depression scale.

Fig. 1 shows the probability of the family intervention being cost-effective on a range of hypothetical threshold values for decision-makers’ WTP for an additional health outcome from a healthcare payer perspective with addition of the participants’ cost. The probability of the family intervention being cost-effective at a WTP of €300 was 99.8% for the MCS, 26.1% for the GAD-7, and 39.9% for the PHQ-9 at 2 months’ follow-up. The probability for the MCS score decreases to 97.2% at 8 months’ follow-up. The probability increases for the GAD-7 score to 70.4% and 52.0% for the PHQ-9 score. Solely on GAD-7 and PHQ-9 scores, there is a notable difference in probability at a WTP of €500 for the 2 months’ follow-up, and these probabilities increase at 8 months’ follow-up.

Evaluating the same WTP thresholds, but only from the healthcare payer perspective, did not have large impact on the probabilities for the family intervention being cost-effective. At a WTP of €300 there was a probability of 99.8% for the MCS, 27.3% for the GAD-7, and 40.5% for the PHQ-9 at 2 months’ follow-up. For a WTP of €500 the probability was 99.9% for the MCS, 57.8% for the GAD-7, and 75.2% for the PHQ-9 at 2 months’ follow-up (Appendix S3).

The sensitivity analyses for the pessimistic and optimistic scenarios did not show notable change for the probability of the 3 outcome measures (see Appendix S2).

## DISCUSSION

This study examined possible predictors of treatment response to the family intervention for the MCS score at 2 time-points. The predictor analyses showed negligible effect sizes of participant characteristics on treatment outcomes at both 2 and 8 months’ follow-up, suggesting that the intervention was similarly effective across different groups, with only a small effect size of injury type on the change in MCS scores at 8 months’ follow-up. Furthermore, we evaluated the cost- effectiveness of the family intervention, which showed a high probability of being cost-effective compared with PEG from the perspective of the healthcare payer and participants’ cost. The highest probability score was found for the outcome measure MCS, indicating this score is the more economically attractive measure at lower WTP thresholds compared with GAD-7 and PHQ-9.

The predictor analyses indicated that the benefits from the family intervention did not differ much in relation to type of injury, time since injury, delivery format, and the relationship of the participants. This indicates that the family intervention has a broad applicability across injury groups, family members, and format of delivery. Individuals with ABI and their family members reported lower gains on the MCS compared with SCI at 8 months’ follow-up, constituting a small effect size. This exploratory analysis highlights the need for future research to investigate the impact of the type of injury on the effect of rehabilitation interventions. A study by Scholten et al. (34), predicting psychological distress among individuals with SCI and ABI and their significant others, found that individuals with SCI and their significant others reported higher levels of psychological distress at baseline and follow-up compared with individuals with ABI and their significant others (34). Despite these differences, the study concluded that the levels of distress are largely consistent across both groups.

In accordance with our findings, a study by Ymer et al. (35) found no effect of time since injury as a predictor of the treatment effect for cognitive behavioural therapy after ABI. In contrast to this and our findings, a previous study found that a shorter time since injury significantly influenced the treatment effect for cognitive behavioural therapy for sleep disturbance and fatigue after ABI (36). The previous study had a mean time since injury of 4.3 years in comparison with a median of 13 months in our study, suggesting that a longer time horizon for time since injury could affect the treatment effect in rehabilitation studies.

The relationship between ABI or SCI survivor and family member did not predict treatment response, indicating that both individuals with injury and family members did benefit from the intervention. In a previous published qualitative study of family intervention, participants described how the intervention benefited not only the individual with the injury, but indeed also the family members (37). The study found that the family intervention supported the families in strengthening cohesion and managing their changed life situation, among others through new insights on each other’s life situations and better communication (37).

The use of telehealth has been progressing in rehabilitation science and can be a more flexible and sustainable solution for people with ABI and SCI (38, 39). We found no evidence that delivery format influenced the treatment response, which is in accordance with a previous study evaluating cognitive behavioural therapy which found that delivery format did not predict treatment effect (35). The adaptation to telehealth for the family intervention could furthermore have a positive economic impact, as the utilities for the participants’ transportation and time would decrease, as well as some modifications for the delivery of the intervention. This transition will result in a lower cost for the intervention, hence a higher probability for cost-effectiveness assuming equal effect. Moreover, telehealth may contribute to larger participation for families as the online format provides accessibility, making the intervention more accessible for more rural areas.

The cost-effectiveness analysis of the family intervention suggests that the FIG could be cost-effective compared with PEG. From a broader societal perspective, the potential implications of the family intervention may be even more substantial. While this study focused on the healthcare payer perspective, it is important to consider the indirect costs and benefits that may arise outside the intervention, e.g., returning to work after sick leave etc. Previous studies highlighted the high costs associated with ABI and SCI, both for the individual with injury, but also for their closest family members, including costs related to healthcare and social care (12, 13). For example, children of a parent with an injury have a higher probability of long-term sick leave by age 26 as well as early retirement (40). The findings for the RCT on the family intervention indicated a reduction in caregiver burden, which may contribute to maintaining caregivers’ health and their attachment to the labour market as well as reduce their use of healthcare services (9). Consequently, such services could be included in future evaluations.

### Study limitations

The findings of this study should be interpreted in light of several limitations. First, the predictor analyses were conducted with a relatively small sample size, which may limit the statistical power and generalizability of the results. In addition, predictors were examined separately, allowing us to assess only their unique effects rather than potential interactions or combined influences.

Second, the cost analysis incorporated the intervention expenses, but not the potential changes in the utilization and costs of health and social services, due to the short time horizon with only 8 months’ follow-up. Ideally, a long-term and broader perspective would have been beneficial for a more comprehensive cost analysis. Improved quality of life may lead to fewer complications and comorbidities, which can result in a reduced burden on the healthcare system and hence lower costs long-term. Furthermore, a reduction in caregiver burden may allow caregivers to maintain their own health and productivity. The effect analysis has limited generalizability, as it was measured by the MCS, GAD-7, and PHQ-9 scores. To ensure comparison between diagnoses and treatments, a more general outcome such as quality adjusted life years (QALYs) would have been beneficial, as QALYs capture an overall health benefit in a single metric and are widely used in cost-effectiveness and cost-utility analyses.

Third, the PEG was an active control group and the comparison against an active control rather than TAU can complicate the characterizing of cost and health benefit of implementation for a family intervention in routine practice. Furthermore, the incremental cost-effectiveness reflects the delivery of the intervention in the context of a clinical trial, whereas regular clinical practice may have different resource availability and implementation costs, which could affect the overall cost-effectiveness.

### Implications

These results highlight the broad treatment effect of family intervention, as the 4 predictors had no or only little impact on the treatment response. For more applicable results, further research should investigate potential predictors of treatment effect in larger study samples, especially the type of injury, and determine how the predictors correlate with each other. Furthermore, this study determined the probabilities for the family intervention to be cost-effective at various WTP thresholds from a healthcare perspective. For a more comprehensive understanding of costs, future research should include a broader perspective with inclusion of health and social care services.

In conclusion, this study provides insight into the treatment response of the family intervention, with no or only small effect of the type of injury, time since injury, delivery format, and relation of participants. Furthermore, the FIG is likely to be cost-effective relative to PEG for improving health measured by MCS, GAD-7, and PHQ-9 in individuals with ABI, SCI, and their family members.

## Supplementary Material


