# Supplementary material for: PREDICTING EFFECT AND EVALUATING COST-EFFECTIVENESS OF A FAMILY INTERVENTION AFTER ACQUIRED BRAIN OR SPINAL CORD INJURY: A RANDOMIZED CONTROLLED TRIAL
Source: J Rehabil Med. 2026 Feb 11;58:44691. doi: 10.2340/jrm.v58.44691 (PMC12914643; doi:10.2340/jrm.v58.44691)
Supplement: Supplementary file 1 [file JRM-58-44691-s1.pdf]

Supplementary material has been published as submitted. It has not been copyedited, or typeset by Journal of Rehabilitation Medicine

Predicting effect and evaluating cost-effectiveness of a family intervention after acquired brain or spinal cord injury: A randomised controlled trial

Appendix S1: Resource costing for delivery of the family intervention group and the psychoeducational group.

A detailed description of each cost unit for the delivery of the two groups, with the addition of the participants' time use and transportation costs for participating in the study (Table S1.1 and S1.2). A description of each cost item, and the source of the base-case cost, are presented in Table S1.3.

Appendix S2: Sensitivity analysis with two scenarios – optimistic and pessimistic.

Two sensitivity analyses with the scenario of a pessimistic and optimistic approach were conducted to determine uncertainty for the cost analyses. The cost for the health care payer perspective, participants cost, and total cost are presented in Table S2.1 and S2.2. Cost-effectiveness acceptability curves (CEAC) for the pessimistic and optimistic scenarios are presented in Figure S2.1 and S2.2.

Appendix S3: Probability of the family intervention being cost-effective.

Based on CEAC, the probability of the family intervention being cost-effective at a willingness-to-pay threshold ranging from €0 to €1000 for each outcome measure at the health care payer perspective and includes the participants costs (Table S3).

Appendix S1: Resource costing for delivery of the family intervention group and the psychoeducation group

Individuals with acquired brain injury (ABI) or spinal cord injury (SCI) and their family members were randomized to a family intervention group (FIG) or a psychoeducational group (PEG). The FIG consisted of 35 families with 74 individual participants. The PEG consisted of 38 families with 83 individual participants. Each family participated in an introduction session, where they completed baseline questionnaires. Afterwards they were randomly assigned to each intervention arm, with an allocation ratio 1:1. Blinding was not feasible for either the families or the neuropsychologist facilitating the two groups.

Family intervention

Families allocated to the FIG received seven sessions of a duration of 90 minutes each with a neuropsychologist. The family intervention builds on strategies from rehabilitation psychology, cognitive behavioural therapy, and family therapy (1). The participating families had between-session tasks. An overview of each session is presented in the original RCT (2).

Psychoeducation

The families allocated to the PEG was offered a 120-minute psychoeducation session with a trained neuropsychologist. The psychoeducation was administrated to groups of families or individual families. In total, there were 24 psychoeducation sessions.

Participants utilities

The participants' time use was based on registered participating session(s), and time used for transportation. We made the assumption that the family travelled together. The same monetary value to hours in paid employment, as for other unpaid activities was applied (3). Table S1.1 and S1.2 summarises the cost of delivery for the FIG and PEG.

Table S1.1. Description of cost per family for the family intervention group (FIG)

**Input**	**Number (A)**	**Unit cost (B)**	**Total cost (A x B)**	**Per family cost (A x B / n = 35)**
Delivery of the FIG				
1 - 8. session of family therapy	A total of 370.5 hours of session time.<br>Average 7.06 session per family (SD:2.01; min: 1; max: 8)	Psychologist: 69.13 € / hour<br>Research nurse: 68.72 € / hour	22,777.06 €	650.77 € / family
Preparation time	A total of 123.5 hours of preparation.<br>Average of 3.5 hours per family (SD: 1.01; min: 0.5; max: 4)	Psychologist: 69.13 € / hour<br>Research nurse: 68.72 € / hour	8,530.38 €	243.73 € / family
Administration	A total of 13.6 hours for administrating communication with participants.<br>Average of 0.39 hours per family (SD: 0.38; min: 0; max: 1.36)	Research nurse: 68.72 € / hour	934.59 €	26.70 € / family
Materials	Every family received at folder with materials for each session. The folder consisted of 56 printed pages. Total of 1,960 printed papers.	0.13 € / A4	254.80 €	7.28 € / family
Overhead	21.95 % of total delivery costs.		7,133.05 €	203.80 € / family
		Sub-total	39,629.88 €	1,132.28 € / family
Resources for participants				
Transportation	A total of 7,204 km. transportation to and from sessions for each family[a].<br>Average: 206 km / family (SD: 307; min: 0; max: 1,648)	0.47 € / km	3,385.88 €	96.74 € / family
Time use	A total of 979.29 hours the participants used on transportation and time in sessions.	36.51 € / hour	35,753.87 €	1,021.54 € / family

Input	Number (A)	Unit cost (B)	Total cost (A x B)	Per family cost (A x B / n = 35)
	Average: 13.2 hours / participant (SD:5.85; min: 1.66; max: 32.2)			
		Sub-total	39,139.75 €	1,118.28 € / family
		**Total**	78,769.63 €	2,250.56 € / family

Note. SD = Standard deviation; [a] Due to the COVID-19 pandemic some participants received the session only, and therefore they had no transportation to/from session.

Table S1.2. Description of cost per family for the psychoeducational group (PEG)

Input	Description (A)	Unit cost (B)	Total cost (A x B)	Per family cost (A x B / n = 38)
Delivery of the PEG				
Psychoeducation	A total of 48 hours of psychoeducation.<br>Average 0.84 hours / family (SD: 0.37; min: 0; max: 1)<br>All families in the PEG also had 20 min with a research nurse.	Psychologist: 69.13 € / hour<br>Research nurse: 68.72 € / hour	4,272.16 €	110.00 € / family
Preparation time	Total of 35 hours of preparation. The preparation time for each session was 30 min.<br>Average 0.92 hours of preparation / family (SD: 1.01; min: 0.5; max: 1)	Psychologist: 69.13 € / hour<br>Research nurse: 68.72 € / hour	1,894.86 €	49. 86 € / family
Administration	A total of 29.24 hours of administrations of communication with participants.<br>Average: 0.77 hours / family (SD: 0.38; min: 0; max: 2.55)	Research nurse: 68.72 € / hour	2009.37 €	52.88 € / family
Overhead	21.95 % of total delivery costs.		1794.72 €	47.23 € / family
		Sub-total	9971.11 €	262.40 € / family

Input	Description<br>(A)	Unit cost<br>(B)	Total cost<br>(A x B)	Per family cost<br>(A x B / n = 38)
Resources for participants				
Transportation	A total of 3,457 km. Transportation to and from sessions for each family[a].<br><br>Average: 92.1 km / family (SD: 195; min: 0; max: 1184)	0.47 € / km	1,624.79 €	42.76 € / family
Time use	The time the participant used on transportation and time in sessions. A total of 287.72 hours per participant (n = 83).<br><br>Average: 3.47 hour / participant (SD: 2.3; min: 0.66; max: 15.66)	36.51 € / hour	10,504.66 €	276.44 € / family
		Sub-total	12,129.45 €	319.20 € / family
		**Total**	22,100.60 €	581.59 € / family

Note. SD = Standard deviation; [a] Due to the COVID-19 pandemic some participants received the session only, and therefore they had no transportation to/from session.

Table S1.3. Description and source of unit cost

Category	Description	Source
Neuropsychologist	According to the Payroll Data Office for Municipalities and Regions (Kommunernes og Regionernes Løndatakontor) in Denmark, the gross monthly salary for a psychologist employed under the collective bargaining agreement for academic professionals within the regional sector was €366,107.9.<br><br>The hourly salary was estimated based on effective working hours in full-time equivalents, as defined by the Danish Health Technology Council (Behandlingsrådet).	https://www.krl.dk/sirka/sirkaApi/tableApi<br><br>Behandlingsrådet. Vejledning til omkostningsopgørelse [Guidelines for cost estimation]. Danish Health Technology Council. https://behandlingsraadet.dk/arbejdsredskaber
Research nurse	According to the Payroll Data Office for Municipalities and Regions (Kommunernes og Regionernes Løndatakontor) in Denmark, the gross monthly salary for a research nurse employed under the collective bargaining agreement for health professionals within the regional sector was €343,757.9.<br><br>The hourly salary was estimated based on effective working hours in full-time equivalents, as defined by the Danish Health Technology Council (Behandlingsrådet).	https://www.krl.dk/sirka/sirkaApi/tableApi<br><br>Behandlingsrådet. Vejledning til omkostningsopgørelse [Guidelines for cost estimation]. Danish Health Technology Council. https://behandlingsraadet.dk/arbejdsredskaber
Administration	The administration of participant was made by a research nurse and each contact had an estimated time of 10 minutes per contact either by email or phone.	See category, research nurse
Materials	Color printed A4 sheets	
Overhead	Overhead costs are not considered attributable to specific resources. According to Copenhagen University Hospital, Rigshospitalet, overhead costs in the context of research are estimated at 21.95%.	
Transportation	Transportation cost was estimated in accordance with the Danish Health Technology Council (Behandlingsrådet) recommends of transportation costs to be valued using the state tax-free mileage allowance (befordringsgodtgørelse).<br><br>Seven families had no information of address. These family's transportation distance was	https://sktst.dk/nyheder-og-pressemeddelelser/koerselsfradraget-og-befordringsgodtgoerelsen-falder-i-2020<br><br>https://www.kl.dk/media/qwmh5kqs/afstand-til-naermeste-

Category	Description	Source
	estimated to the standard distance to nearest hospital (20 kilometer).	sygehus-fugleflugt-eller-vejafstand.pdf
Productivity	Productivity was assessed as comprising both paid and unpaid work or activities. Consequently, participants' absence from other activities is considered a loss of productivity.<br><br>The productivity loss was estimated in accordance with the Danish Health Technology Council (Behandlingsrådet) by the average pre-tax hourly wage of an employee in Denmark.	https://www.statistikbanken.dk/LONS20<br><br>Behandlingsrådet. Vejledning til omkostningsopgørelse [Guidelines for cost estimation]. Danish Health Technology Council. https://behandlingsraadet.dk/arbejdsredskaber

Appendix S2: Sensitivity analysis with two scenarios – optimistic and pessimistic

The main result for this cost-effectiveness analysis is based on most plausible estimates for the costs parameters included. These are associated with some uncertainty, hence the result for the main result involves some uncertainty. To determine uncertainty of the cost, a scenario sensitivity analysis was made by adjusting the various cost items by ± 25%. Same analysis approach as for the main analysis were made.

Table S2.1. Optimistic health payer cost and participant cost for the family intervention group (FIG) and the psychoeducational group (PEG), per participant

	Mean cost, (SE)		Incremental cost, (95% CI)
	FIG	PEG	$\Delta C^{FIG - PEG}$
Health care payer	€401.64 (16.01)	€90.10 (4.17)	€11.54 (279.1; 344.0)
Participant	€396.69 (23.81)	€109.60 (10.30)	€287.08 (236.2, 337.9)
Total cost	€798.23 (35.23)	€199.70 (12.04)	€598.62 (525.6; 671.6)

Note. SE = Standard error; CI = confidence interval

Table S2.2. Pessimistic health payer cost and participant cost for the family intervention group (FIG) and the psychoeducational group (PEG), per participant

	Mean cost, (SE)		Incremental cost, (95% CI)
	FIG	PEG	$\Delta C^{FIG - PEG}$
Health care payer	€669.40 (26.69)	€150.17 (6.94)	€519.23 (465.2; 573.3)
Participant	€661.14 (39.69)	€182.67 (17.17)	€478.47 (393.7, 563.2)
Total cost	€1330.54 (58.72)	€332.84 (20.07)	€997.70 (876.1; 1119.3)

Note. SE = Standard error; CI = confidence interval

Figure S2.1. Cost-effectiveness Acceptability Curves for the optimistic health payer approach, at two and eight months

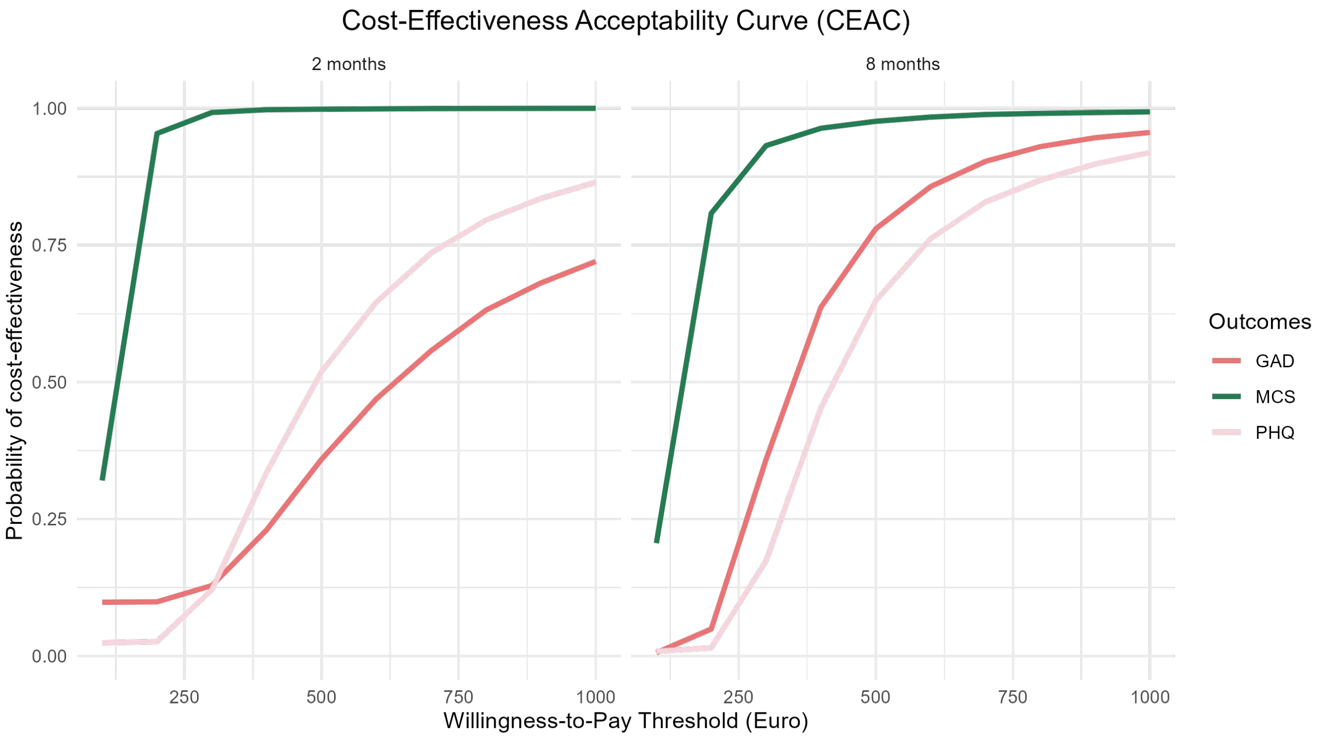

Figure S2.2. Cost-effectiveness Acceptability Curves for the pessimistic health payer approach, at two and eight months

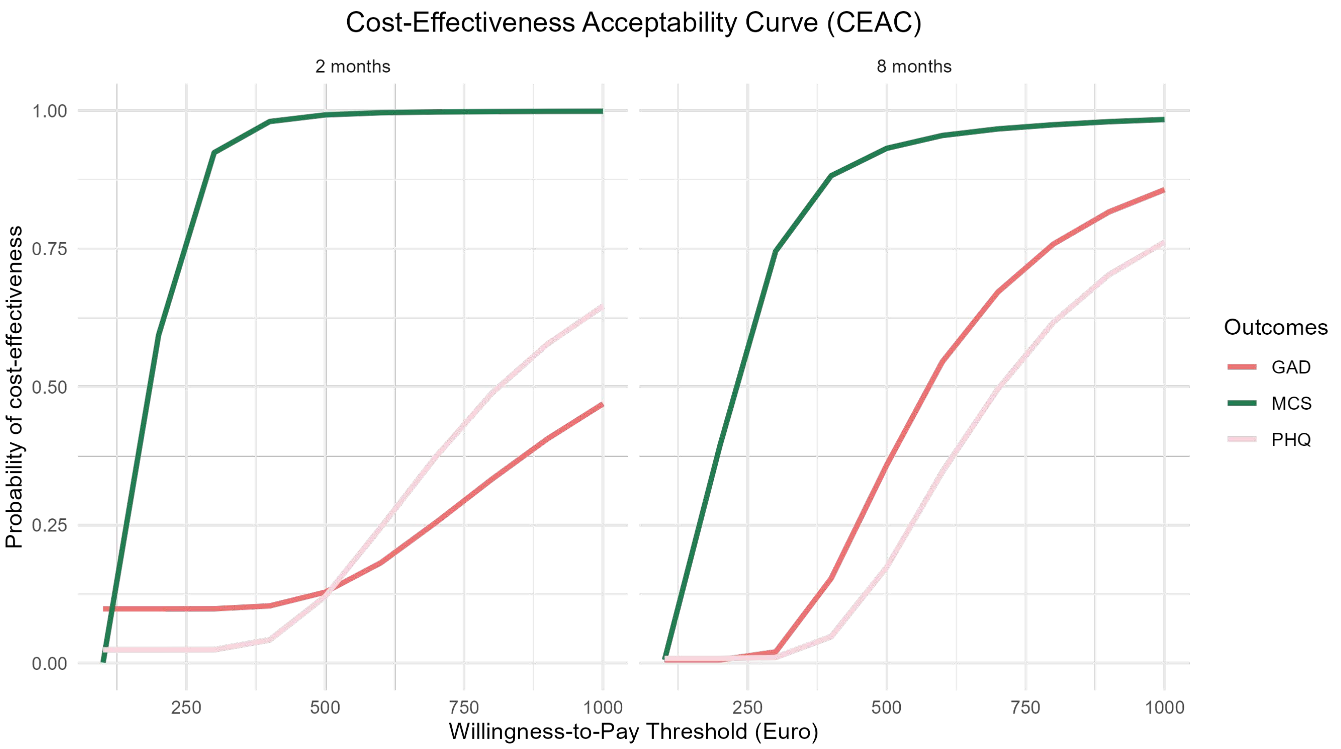

Appendix S3: **Probability of the family intervention being cost-effective**

Table S3. Probability for the family intervention being cost-effective based on acceptability curves, from the perspective of the health care payer and the addition of the participants costs

	Two months		Eight months	
WTP thresholds	Health care payer + participants cost	Health care payer	Health care payer + participants cost	Health care payer
MCS				
€0	0.00%	0.01%	0.41%	0.34%
€100	80.70%	79.98%	59.50%	60.09%
€200	99.01%	99.00%	92.69%	93.04%
€300	99.81%	99.75%	97.15%	97.56%
€400	99.91%	99.88%	98.40%	98.55%
€500	99.96%	99.94%	98.93%	99.08%
€600	99.97%	99.97%	99.28%	99.39%
€700	99.97%	99.97%	99.40%	99.54%
€800	99.97%	99.98%	99.47%	99.63%
€900	99.97%	99.98%	99.53%	99.67%
€1000	99.97%	99.99%	99.60%	99.71%
GAD-7				
€0	9.85%	9.83%	0.59%	0.58%
€100	9.85%	9.83%	0.68%	0.65%
€200	11.86%	12.17%	31.95%	32.75%
€300	26.09%	27.30%	70.37%	69.95%
€400	43.77%	44.83%	84.98%	85.53%
€500	56.24%	57.77%	91.26%	91.51%
€600	64.92%	66.60%	94.28%	94.47%
€700	71.59%	72.41%	95.94%	95.99%
€800	76.07%	76.66%	97.07%	96.98%
€900	79.56%	80.11%	97.71%	97.56%
€1000	82.40%	82.68%	98.21%	97.99%
PHQ-9				
€0	2.20%	2.42%	0.81%	0.68%
€100	2.20%	2.42%	0.81%	0.68%
€200	10.05%	10.25%	14.70%	15.02%
€300	39.85%	40.47%	51.98%	52.68%
€400	62.26%	62.60%	73.87%	73.71%
€500	74.95%	75.24%	83.92%	84.31%
€600	82.07%	82.44%	89.19%	89.80%
€700	86.25%	86.79%	92.12%	92.69%
€800	88.90%	89.65%	93.84%	94.37%
€900	90.94%	91.45%	95.08%	95.55%
€1000	92.54%	92.95%	95.92%	96.35%

Note. WTP = Willingness to pay; MCS = Mental Component Summary; GAD-7 = Generalized Anxiety Disorder; PHQ-9 = Patient Health Questionnaire

Reference

1. Stevens LF, Lehan T, Durán MAS, Plaza SLO, Arango-Lasprilla JC. Pilot Study of a Newly Developed Intervention for Families Facing Serious Injury. Top Spinal Cord Inj Rehabil 2016; 22(1): 49–59. DOI: 10.1310/sci2201-49

2. Soendergaard PL, Arango-Lasprilla JC, Wolffbrandt MM, Dornonville de la Cour FL, Biering-Sørensen F, Norup A. Investigating the Effectiveness of a Family Intervention after Acquired Brain or Spinal Cord Injury: A Randomized Controlled Trial. J Clin Med 2023; 12(9): 3214. DOI: 10.3390/jcm12093214

3. Krol M, Brouwer W. How to estimate productivity costs in economic evaluations. Pharmacoeconomics 2014; 32(4): 335–344. DOI: 10.1007/s40273-014-0132-3
